# Breast cancer preventive practices and associated factors among reproductive age women in Wadila District, North East Ethiopia: community based cross-sectional study

**DOI:** 10.1186/s12885-024-12613-y

**Published:** 2024-07-15

**Authors:** Zemene Damtie, Niguss Cherie, Melaku Mekonnen Agidew

**Affiliations:** 1 Department of Public Health, Zemen Postgraduate College, Dessie, Ethiopia; 2 School of Public Health, College of Medicine and Health Sciences, Wollo University, Dessie, Ethiopia; 3 Department of Biomedical Sciences, College of Health Sciences, Debre Tabor University, Debre Tabor, Ethiopia

**Keywords:** Breast cancer, Reproductive age women, Knowledge, Preventive practice, North East Ethiopia

## Abstract

**Background:**

Cancer is one of the leading causes of morbidity and mortality worldwide. Among all cancer types, breast cancer stands out as the most common and is characterized by distinct molecular characteristics. This disease poses a growing public health concern, particularly in low and middle-income countries where it is associated with high mortality rates. Despite these challenges, there is a paucity of data on breast cancer preventive practices and associated factors among reproductive-age women in Wollo, Ethiopia. Hence, this study aimed to evaluate the level of breast cancer awareness, preventive practices, and associated factors among women of reproductive age residing in Wadila district, Wollo, Ethiopia in the year 2022.

**Method:**

A cross-sectional community-based study involving 352 women of reproductive age in Wadila district was carried out between May and June 2022. Participants were selected using a systematic random sampling technique, and data analysis was conducted using Statistical Package for Social Science (SPSS) version 23 software. Logistic regression analysis was utilized to determine the odds ratio for variable associations, with statistical significance set at *p* < 0.05.

**Result:**

The prevalence of breast-examination among women of reproductive age was determined to be 40.1% (95% Interval [CI]: 34.94–45.18). Factors such as educational status (Adjusted Odds Ratio [AOR]: 0.28, 95% CI: 0.13–0.6), income (AOR: 0.19, 95% CI: 0.11–0.33), and family history of breast conditions in reproductive-age women (AOR: 1.90, 95% CI: 1.08–3.34) were significantly linked to the practice of breast self-examination in this population.

**Conclusion:**

The study highlighted a decline in regular breast self-examination among women of reproductive age. It revealed that the reduced frequency of regular breast self-examination was a prevalent concern among women in this age group and the broader community. Educational level, monthly income, and family history of cancer among women of reproductive age were identified as significant factors linked to the practice of regular breast examination.

## Introduction

Cancer remains a significant global health challenge, contributing to high rates of illness and death worldwide. Data from the International Agency for Research on Cancer (IARC) indicates that there were approximately 14 million new cancer cases reported in 2012 [1]. Among these cases, breast cancer stands out as the most commonly diagnosed malignant tumor in women and the second leading cause of female mortality globally [2]. This disease is characterized by distinct clinical, pathologic, and molecular features [2, 3].

The prevalence of breast cancer poses a growing public health concern, particularly in low and middle-income countries where it is a leading cause of mortality [3]. Despite efforts to address risk factors and promote prevention, there is no population or woman globally who is truly immune to the possibility of developing breast cancer [2]. The incidence of breast cancer has now surpassed that of lung cancer, with an estimated 2.3 million new cases worldwide [4].

In Lebanon, the median age at which breast cancer patients present is 49 years, underscoring the importance of early detection and intervention [4]. While breast cancer was historically more common in developed nations, its impact has now spread to developing countries, posing a significant threat to public health [3]. Alarmingly, nearly half of all breast cancer cases and over half of related deaths occur in low and middle-income countries, highlighting the urgent need for improved prevention and treatment strategies on a global scale [3].

The number of new cancer cases in Ethiopia is on the rise, with breast cancer being the most common type of cancer among women, accounting for 33% of female cancers and 23% of all cancers in the country. Breast cancer has emerged as a leading cancer among Ethiopian women, with an age-adjusted incidence rate of 41.8 per 100,000 women. In 2012, an estimated 12,956 new cases of breast cancer were reported in the country [5]. A study conducted at Tikur Anbessa Specialized Hospital Radiotherapy Center revealed that breast cancer is the second most prevalent malignancy, representing 27.8% of all cancer cases referred to the hospital. The healthcare system in Ethiopia faces challenges in managing the prevention and treatment of cancer due to limited resources and a focus on addressing communicable diseases like HIV, malaria, and tuberculosis [6].

Raising awareness about breast cancer plays a crucial role in determining when patients seek medical help for the disease [7]. Studies indicate that women’s knowledge and practices related to breast cancer prevention and management can greatly influence their decision to seek medical assistance [8]. Utilizing screening methods for early detection of breast cancer is a key strategy in reducing mortality rates associated with the disease [9]. Detecting breast cancer at an early stage allows for prompt treatment before it spreads, leading to better outcomes. Achieving early diagnosis involves enhancing cancer awareness, encouraging active participation in healthcare, ensuring accurate clinical evaluations, obtaining proper pathological diagnosis, staging the disease accurately, and enhancing access to healthcare services [10].

Timely identification and diagnosis of cancer play a vital role in enhancing treatment success rates, emphasizing the significance of promoting awareness about symptoms of the disease. It is essential to grasp the extent of understanding and preventive measures concerning breast cancer among women of childbearing age to minimize its effects. In Ethiopia, there is a dearth of extensive information on breast cancer awareness and preventative actions in this specific group. The insufficient research conducted in this domain underscores the necessity to address this knowledge disparity. Hence, our research endeavors to evaluate the awareness, preventative practices, and influencing factors related to breast cancer among women of reproductive age in Ethiopia.

## Methods and materials

### Study area and period

The research was carried out in Wadila district, an administrative woreda located in the North Wollo zone between April 22 and June 20, 2022. The district is situated 120 km northeast of Woldia and 320 km northeast of Bahir Dar. It is home to a population of 136,407 individuals spread across 24 rural and 2 urban kebeles. Of this population, 50.2% are female, and there are 32,165 individuals of reproductive age. The district is served by six health centers and one district hospital providing essential healthcare services to the community.

### Study design

We utilized a community-based cross-sectional study design to examine reproductive-age women living permanently in the Wadila district. The sample consisted of reproductive-age women residing in Wadila district throughout the study duration.

### Eligibility criteria

We included all women of reproductive age who lived permanently in the Wadila district and those who were present during the data collection. Women of reproductive age with visual, hearing, or speech impairments, as well as those with a history of breast cancer, were excluded from the study.

### Sample size determination and sampling technique

#### Sample size determination

The sample size was determined using the single population proportion formula, assuming the proportion of females who practiced self-breast examination (SBE) were.

68%^12^,with a 95% confidence interval and 5% margin of error.

$${\rm N} = \frac{{{{({\rm Z}a/2)}^2} \times ({\rm p})({\rm q})}}{{{{\rm{d}}^2}}}$$

Where;

n = sample size required.

α = the level of significance (5%).

Z*a*/2 = the value of Z (the standard normal distribution value) at the selected level of.

Significance = 1.96.

p = proportion of females who practiced SBE were 68%,

$${\rm{d }} = {\rm{ Margin}}\,{\rm{of}}\,{\rm{error}} = 0.05$$

$${\rm{n}}\,{\rm{ = }}\frac{{{\rm{(1}}{\rm{.96)2}} \times {\rm{(0}}{\rm{.68}} \times {\rm{0}}{\rm{.32)}}}}{{{{(0.05)}^2}}}$$

*N* = 335. Adding 5% non-response rate, the sample size becomes 352.

#### Sampling technique

There are 26 kebeles in the district and five of them were selected and included in the study by using random sampling technique. The numbers of households from each kebeles were determined based on their population size using a systematic random sampling technique with independent sampling intervals (kth value) applied to each kebele. Here, the sample size was also allocated proportionally according to the number of households in each selected kebele. Only one individual women in each household was selected and included in this study. If more than one eligible woman were found in a single household, we selected only one of them by lottery method.

### Data collection procedure

The data was collected by using a structured and self-administered questionnaire that was designed by reviewing previous similar peer-reviewed published studies [12–14] and administered by interviewers. The questionnaire was pre-tested before the actual data collection and evaluated by research team to assess the content validity. The questionnaire consisted of all the variables that can meet the objectives of the study which are related to socio-demographic characteristics, substance use, known medical illnesses, knowledge, and practice regarding breast cancer prevention practice and early detection of risk factors.

### Operational definition

#### Good practice of breast cancer prevention

Those who carried out SBE practice a week after each menses using their palm and middle three fingers, or at least one clinical breast examination, or at least one mammography checkup in their life time [15].

#### Good knowledge about breast cancer early detection methods

We considered our participants as having good knowledge if they answered more than or equal to the mean score of knowledge assessment questions in our study [12].

#### Knowledge of women on breast cancer risk factors

This part was assessed by using reference guidelines of the American Cancer Society [15]. Our participants were said to have good knowledge if they answered more than half of the questions assessing their knowledge on risk factors of breast cancer.

### Study variables

#### Dependent variables

Knowledge and level of practice of breast cancer prevention methods were considered as dependent variables while independent variables include socio-demographic factors (age, religion, marital status, level of education, occupation), life style factors (exercise, diet, substance use), reproductive and medical history (pregnancy status, family history of breast cancer, history of any breast disease, history of contraceptive, and comorbidity).

### Data quality control

To maintain data quality, three data collectors who were health professionals (clinical nurses) were selected based on their experience in data collection and they were trained for 2 days on the objectives of the study, interview techniques, and ethical considerations during data collection. The questionnaire was properly developed by the research team by reviewing different kinds of previous peer-reviewed published articles [12] according to the specific objectives. The questionnaire was first prepared in the English language and translated into the local “Amharic” language to facilitate communication. The collected data was revised and checked for mistakes, legibility of handwriting, completeness, and consistency by the researchers daily during data collection before data entry and statistical analysis. Any mistake or ambiguity was cleared on the spot.

### Data analysis procedure

Data analysis involved checking for completeness, coding, entry into Epi Data version 3.1, and export to Statistical Package for Social Science (SPSS) Version 23 for summarization and analysis. Descriptive statistics such as frequencies, means/medians, and proportions were used. Variables with a *p*-value < 0.25 in bivariable logistic regression were included in a multivariable model to identify independent associations. Adjusted odds ratios with 95% confidence intervals were reported, with a significance level of *p* < 0.05.

### Ethical clearance

Ethical approval was obtained from the Zemen Post Graduate Coordinating Program Ethics Review Committee, with cooperation letters secured from the Wadela District Health Office and kebele administration offices. Informed consent was obtained from each study participants before data collection, ensuring confidentiality by removing any identifiable information. For those study participants who were less than 18 years of age, the informed consent was obtained from their parents or care givers.

## Results

### Sociodemographic characteristics of the reproductive-age women

A total of 23.9% of women in the study fell within the age group of 30–34 years, with 57.4% residing in rural areas. Additionally, 61.1% of the women identified as followers of the Orthodox religion, while 84.1% belonged to the Amhara ethnic group. Among the participants, 35.2% had completed secondary education, and 27.8% were engaged in farming activities (Table 1).

**Table 1:** Sociodemographic characteristics of reproductive-age women in Wadila District, Amhara Region, Ethiopia, 2022

Variables		Frequency	Percent
Age category	15–19	75	21.3
	20–24	78	22.2
	25–29	72	20.5
	30–34	84	23.9
	≥ 35	43	12.10
Residency	Rural	202	57.4
	Urban	150	42.6
Religion	Orthodox	215	61.1
	Muslim	83	23.6
	Others	54	15.3
Ethnicity	Amhara	296	84.1
	Others	56	15.9
Marital status	Single	138	39.2
	Married	146	41.5
	Divorced	46	13.1
	Widowed	22	6.2
Educational status	Illiterate	98	27.8
	Primary education	70	19.9
	Secondary education	124	35.2
	College/University	60	17.1
Occupational status	Employee	65	18.5
	Merchant	67	19.0
	Farmer	98	27.8
	Housewife	64	18.2
	Labour worker	49	13.9
	Jobless	9	2.6
Income	Below poverty line	264	75
	Above poverty line	88	25

All the variables are continuous so that they are presented as number and percent. Others include Protestants and Catholics for religion and Oromos and South Nations, Nationalities, and Peoples for ethnicity

### Medical history and lifestyle conditions of reproductive-age women

Regarding the health history, 13.6%, 10.2%, and 9.9% of the participants reported a history of hypertension, diabetes, and other chronic diseases, respectively. Furthermore, 3.7% of them had a family history of breast cancer, and 13.9% had experienced other breast diseases. Substance abuse history was also reported by 15.6% of the subjects, while 33.2% engaged in regular physical exercise (Table 2).

**Table 2:** Medical history and lifestyle conditions of reproductive-age women in Wadila District, Amhara Region, Ethiopia, 2022

Variables		Frequency	Percent
Hypertension	Yes	48	13.6
	No	304	86.4
Diabetics	Yes	36	10.2
	No	316	89.8
Other chronic diseases	Yes	35	9.9
	No	317	90.1
Family history of breast cancer	Yes	92	26.1
	No	260	73.9
Other breast diseases	Yes	49	13.9
	No	303	86.1
Substance abuse	Yes	55	15.6
	No	297	85.4
Regular physical exercise	Yes	117	33.2
	No	235	66.8

All the variables are continuous so that they are presented as number and percent

### Knowledge of reproductive-age women about breast cancer, its risk factors, and symptoms

The study revealed that 65.3%, 86.6%, and 69.0% of participants had poor knowledge about breast cancer, its risk factors, and symptoms, respectively. Only 40.1% (95% CI: 34.94–45.18) practiced breast checkups, with just 91.49% of them performing self-examinations. The initiation of breast examination typically began at age 30 and above for 39.0% of women (Table 3).

**Table 3:** Assessment of knowledge of reproductive-age women about breast cancer, its risk factors, and symptoms in Wadila District, Amhara Region, Ethiopia, 2022

Variables		Frequency	Percent
Knowledge about breast cancer	Good	122	34.7
	Poor	230	65.3
Knowledge on risk factors of breast cancer	Good	47	13.4
	Poor	305	86.6
Knowledge on symptoms of breast cancer	Good	109	31
	Poor	243	69
Breast -examination	Yes	141	40.1
	No	211	59.9
Examiner	Self	129	91.49
	Health provider	12	8.51
Starting age (year)	< 20	49	34.8
	20–30	37	26.2
	> 30	55	39.0
Frequency of examination	Weekly	7	5.0
	Monthly	15	10.6
	Occasionally	49	34.8
	During abnormality	70	49.6

All the variables are continuous so that they are presented as number and percent

### Factors associated with regular breast examination among reproductive-age women

Although there are many and different factors associated with regular breast self-examinations, the analysis of our study identified that educational status, occupational status, income level, knowledge about risk factors of breast cancer, family history of breast cancer, and history of other breast diseases were variables with *p* < 0.25 in bivariate analysis and fitted into multiple logistic regression model to see independently significant predictor variables for regular breast self-examinations by using AOR with 95% CI. However, the multivariate logistic regression analysis found that only educational status, level of income, and family history of breast cancer were independent significant predictors for regular breast self-examinations among reproductive-age women. Women with college/university education were more likely to undergo regular breast self-examinations compared to those who were illiterate [AOR: 95% CI: 0.28 (0.13–0.6)]. Conversely, individuals with an income above the poverty line were more likely to have regular breast examinations [AOR: 95% CI: 0.19 (0.11–0.33)]. Contrary, women with a family history of breast cancer were less inclined to have regular breast examinations compared to those without such a history [AOR: 95% CI: 1.90 (1.08–3.34)] (Table 4).

**Table 4:** Assessment of factors associated with regular SBE among reproductive-age women in Wadila District, Amhara Region, Ethiopia, 2022

Variables		Breast self-examination		COR (95% CI)	AOR (95% CI)	*p*-value
		Yes	No			
Education	Illiterate*	22	76	1 0.41 (0.20–0.81)	1 0.28 (0.13–0.89)	**0.001**
	Primary	29	41			
	Secondary	53	71			
	College/above**	25	35			
Occupation	Employee**	46	19	1 1.98 (0.98–2.97)	1 0.47 (0.12–1.55)	0.097
	Merchant	21	46			
	Farmer	30	68			
	Housewife	18	46			
	Labour worker	10	39			
	Jobless*	4	5			
Income	< Poverty*	74	190	1 0.23 (0.14–0.39)	1 0.19 (0.11–0.33)	**0.000**
	> Poverty**	55	33			
Knowledge on risk factors of breast cancer	Good**	19	28	1 5.12 (2.81–9.04)	1 0.99 (0.48–2.64)	0.159
	Poor*	110	195			
Family history	Yes**	27	65	1 1.55 (0.93-26)	1 1.9 (1.08–3.34)	**0.026**
	No*	102	158			
Other breast diseases	Yes**	12	37	1 2.62 (1.19–8.73)	1 11.6 (7.59–18.93)	1.002
	No*	117	186			

Those categories having variables marked by single strikes (*) are reference categories while those categories having variables marked by double strikes (**) contain significant variables to which the COR and AOR was done

## Discussion

The study revealed that the overall rate of lifetime regular breast examination among reproductive-age women in Wadila District was 41.1% (95% CI: 34.94–45.18). Among those who underwent regular breast examinations, 91.49% practiced SBE. This percentage is concerning given the high prevalence of breast cancer, a leading chronic disease that requires early detection and intervention [16]. These findings align with a previous study conducted among reproductive-age female healthcare workers in Iran, which also reported low consistency in SBE practices among employed women in health organizations [17]. Similarly, a study among reproductive-age female students at Debre Berhan University found that only 28.3% of participants had performed SBE [18]. These results underscore the importance of raising awareness and promoting regular breast examinations, particularly SBE, among reproductive-age women to improve early detection and management of breast cancer.

However, the results of this study differ from those of a previous study conducted among female tertiary students in Ghana, which reported that 73.0% of the participants had practiced SBE at least once [19]. The discrepancy in findings may be attributed to the inclusion of both educated and non-educated women of reproductive age in our study, who may be less inclined to engage in breast examinations compared to their educated counterparts. Additionally, our findings contrast with those of a prior study in Ethiopia, where the prevalence of SBE was only 4.3%, significantly lower than our observed rate [14]. This inconsistency might be due to the possibility that women in our study had greater awareness of breast cancer, as it is a rapidly increasing health concern within the community [3] and a topic have been widely discussed from the previous to the current time, leading to increased recognition and engagement with breast examination practices.

The educational status of women was identified as a significant factor influencing the frequency of SBE over their lifetime. Multiple logistic regression analysis in this study revealed that reproductive-age women who had completed College/University were more likely to engage in SBE compared to those who were illiterate. This finding aligns with research conducted among female tertiary students in Ghana, which showed that fourth-year students were more likely to practice SBE than first-year students [19]. Additionally, our results are consistent with a previous study in Sweden that examined factors affecting women’s engagement in SBE [20]. A study in Addis Ababa, Ethiopia, also found a statistically significant correlation between women’s educational status and SBE [14]. However, our findings contrast with a study among reproductive-age female students where only about 3.5% reported engaging in lifetime breast examinations [18]. Similarly, a study in Southern Ethiopia reported an overall magnitude of lifetime breast examination at 34.3%, which differs from our findings [21]. These discrepancies may be attributed to differences in the academic status of participants across the various studies.

The income level of the study participants emerged as a significant factor influencing the adoption of SBE. Our study revealed that reproductive-age women who were above the poverty line were more likely to engage in SBE compared to their counterparts. This finding is corroborated by a previous study conducted in Sweden [20]. Additionally, our results align with other studies conducted worldwide among reproductive-age women [22–24]. The findings of our study are also in line with a previous study conducted in Ethiopia [14].

Another factor significantly associated with the practice of SBE among reproductive-age women was family history of cancer. This finding is inconsistent with findings from various previous studies [22, 25]. Besides, our results contrast with the a previous study conducted among reproductive-age females in Mexico, which reported no significant difference in SBE practice between women with and without a family history of breast cancer [26]. This discrepancy may be attributed to the genetic nature of cancer, as it is known to be hereditary [27]. The genetic diversity of the reproductive-age women in our study compared to those in Mexico may account for the observed inconsistency.

### Strength and limitation of the study

The study’s key strength lies in its originality, as it sheds light on the assessment of breast cancer knowledge level, its preventable practices, and associated factors among reproductive age women in Ethiopia. This serves as a foundational reference for future in-depth studies. However, the study encountered some limitations like it was difficult to take the responses of the participants since the residents of the community both in the rural and urban areas were ashamed to participate in face-to-face interview and provide the appropriate responses for some questions. Thus, some sort of social bias might be present even though the survey was done anonymously by arranging the female interviewer for those female study participants (reproductive-age women. The other limitation of this study was that it was a cross-sectional study, which could not determine the causal relationships.

## Conclusion

In the present study, the magnitude of overall regular breast examination and particular SBE among reproductive-age women was subsided. This study found that the diminished regular breast examination was a widespread issue of reproductive-age women and the community at large as women are parts of the community. The current study found that there were different factors significantly associated with regular breast examination including educational status, monthly income, and family history of cancer among reproductive-age women.

## Data Availability

The datasets used and/or analyzed during the present study are available from the corresponding author on reasonable request.

## Abbreviations

IARC: International Agency for Research on Cancer
LMICs: Low and Middle-Income Countries
SBE: Self-breast examination
